# The Enamel

**Published:** 1874-06

**Authors:** J. F. Hassel


### THE ENAMEL.

*Is it good practice to leave the Dentinal Tubuli open?*

BY DR. J. F. HASSEL.

We are told that after a tooth is fully shaped, before it has yet made its appearance above the gum, that the crowning act of nature is to completely encase it in a substance capable of resisting all materials, of whatever nature, proper to be taken in the mouth. In the perfect state the enamel contains but an extremely minute quantity of animal matter. The enamel is almost literally a mineral, having only 3.59 parts of animal matter in 100. We find, too, that where, as in ordinary bone the phosphate of lime constitutes only 54 parts in 100, in the enamel of the teeth it is nearly 90 parts in 100. While the carbonate of lime in bone amounts to 9.41, in the enamel of teeth it is only 4.37. It will be observed that in bone or dentine there are 54 parts only in a hundred and in the enamel of the teeth 90 parts in 100. With a few and savage like passes of the file, (and allow me to say, that the use of the file on the teeth is of African origin) is exposed to view the dentine, beneath with its open tubes and intertubular spaces. When the walls of the cavity of the pulp of a tooth is regarded with sufficiently high magnifying power, it is seen to be perforated by numerous small tubes separated by numerous narrow interspaces, these are the openings of the dental tubes. They are described by microscopists as having distinct parietes of a harder material than the intertubular tissue. These tubuli commence on the walls of the pulp cavity and radiate in a wavy course through every part of the dentine to its periphery, near the masticating surface of the crown of a tooth; they have nearly a vertical direction and towards the approximal surface a horizontal direction. It is the opinion of some that these tubes contain an organic earthy matter in granular masses. Some ascribe to the dental tubes and cells, the office of distributing to the tooth a nutritive fluid secreted by the surface of the pulp or nerves. That the dentinal part of a tooth is vascular and capable of being injected with red blood is now well established. Owen, Schwan and others entertain pretty much the same opinion. In sections of teeth which have been dried, the tubes become very distinct, and we may sometimes on adding a colored fluid to the preparation, when subjected to a good microscope, observe the tubes becoming gradually filled, etc. I believe the conclusions of Tomes, now are, that each dentinal tube is permanently occupied by a soft fibril which after passing from the pulp into the tubes follows its ramifications. He furthermore says, the presence of soft tissue would not, however, hinder the slow passage of fluids, and that fluids do pass through or by the sides of the fibrils is rendered probable by the fact, that the latter are capable of undergoing structural change at the parts furthest removed from the pulp. I have introduced the above to show, according to the best information we have, that the dental tubuli do contain a fluid, and that they can be injected.

The air through the instrumentality of the oxygen which is one of its component parts, is about the most powerful agency of destruction furnished by the whole armory of nature; it corrodes the steel bars by which the stones are clamped together; it causes the gradual decay of the timber of which the roofs of buildings are usually constructed, so that we seldom find any traces of them in the more ancient remains which have come down to us. Thus the great principle of organic life becomes also in its inevitable and eternal action the great principle also of decay and dissolution. Then follows what we may term the unintentional or accidental agencies of living things. As soon as the walls and pediments and columns of a statue or a temple have lost their polished surface, or, if you please, its enamel through the operation of the chemical influences, the seeds of lichens and mosses and other parasitic plants, which are constantly floating in the atmosphere, settle in the roughnesses, grow, decay and decompose, form soil, attract moisture, and are followed by other and stronger plants, whose roots force their way into the crevices, thus formed by chemical action, and end by wrenching asunder the damaged and disintegrated blocks of marble. The animal creation succeeds the vegetable and aids its destructive operations, the fox burrows, the insect bores, the ant saps the foundations of the building, and thus by a series of causes, all of them in the ordinary and undying course of nature, the most magnificent edifices ever raised by the genius, the piety and the industry of man are brought to an end, as by fixed and irreversible decree.

Is it not so with the pearly citadel? Let us see. We have first the chemical effect, thereby removing the enamel or polished surface, then we have the vegetable or fungus settling in the rough and open mouths of the dentinal tubuli, causing decomposition and decay, lastly, the animal creation, which completes the work of destruction; you observe that I give three prime causes, for the destruction of the teeth, all three of which perform their particular part of destruction.

After the enamel of a tooth is removed by the action of acids or by the hand of man with the file, then it is that the elements of the fungi glide easily into the interior of the canaliculi, which they dilate and thus favor the passage of the animal parasite into the more vital and deeper parts, these same elements can not penetrate a compact enamel. I might modify this remark by saying, at least, they enter more slowly and only when the elements which form it have been greatly changed by the actions of acids. Wherever a loss of substance has occurred at the surface of the dentine, then the elements of the fungus pass into the interior of the dental tissues and produce by their extensions, especially in the dentine, effects of softening and destruction. It is this softened and diseased condition that is so inviting to the animal creation, which I shall now notice.

*The animal cause.* That we have oral parasites belonging to the animal kingdom there is no question of doubt. Leber and Rottenstein, have seen in their examinations with the microscope in teeth that have been deprived of the enamel, mark well the language, in teeth that have been deprived of the enamel, the dentinal tubuli filled with Leptothrix Buccalis parasites belonging to the animal kingdom. Leber and Rottenstein are not the only authority for Leptothrix Buccalis being found in the oral cavity. We have good evidence of their presence nearer home. Prof. Judd has given much time and attention to this subject, he has discovered that some of them, (Leptothrix Buccalis) have feet which they plant against a support and then retract with the greatest possible velocity, watching the motion of one of them he had seen it tear its foot completely off.

The above authority proves conclusively to my mind, that the animal kingdom takes no part in the work of destroying a tooth, until the dental tubes are exposed to their action. There is much evidence I think that could be brought to prove beyond any sort of doubt, that the moment the enamel is removed thereby exposing the dentine and tubes, that moment it falls a prey to animal parasites.

I will relate a case that happened under my observation a patient, called, for the purpose of having something (as he called it) removed from between his teeth, he directed my attention to the space between first superior molar and second bicuspid, left side. I examined as directed, but could see nothing wrong, and so informed him. My diagnosis did not satisfy him, and while leaving my office, remarked, there is something wrong. It was not long before he called again, this time telling me it felt like a hair or piece of stick, I made the second examination this time with more care, assisted with a good magnifying glass, which very plainly revealed, a small piece of wood, the space between the teeth V shape, tubuli badly exposed, in each tooth a small filling of gold, gold entirely surrounded by a thousand and one open tubes. The piece of wood was sticking in mesial surface of molar tooth, midway between the filling and enamel, it was undoubtedly forced into one of the larger tubes while in the act of picking the teeth; he afterwards informed me that he was in the habit of using wooden tooth-picks for the purpose of cleaning his teeth. I discovered, too, that the surface of the dentine was very rough and exceedingly painful to touch of instrument, etc. Are not all such conditions as the above the work of Leptothrix Buccalis? I think so.

The pulp of a tooth is connected in the fully formed tooth with the great centers of life by means of a cord so minute that the canal through which it passes into the root of a tooth can scarcely be seen with the naked eye where it commences, and yet this hair like cord contains an artery vein and nerve, in view of the above facts, it is possible that this spicula of wood was forced into one of the larger tubes, while in the act of cleansing the teeth.

I will relate one more case, out of many that have come under my treatment. A patient called to learn, if possible, the cause of her tooth turning dark. This tooth, a second inferior bicuspid, left side, distal surface, presented a similar condition to the first case, a small gold filling entirely surrounded by the mouths of dental tubuli considerably dilated. My response was, that by refilling, and medicating, the cause of objection would be removed. My suggestion met with her approbation. To test the truthfulness of the dentinal tubuli being capable of permitting secretions to pass from the oral cavity into the body of the tooth, and bringing about this condition of things, I took the precaution to apply rubber dam, so as to prevent all possibility of the secretions entering into the cavity of the tooth. Removal of the filling revealed the startling fact that the tubes were full of stinking matter. Litmus paper sounded the alarm of acid reaction. All such cases prove to my mind that when the enamel is removed there are tubes left exposed that will certainly make mischief. Calcification comes to the rescue and would seem to be an impassable barrier. Dr. Arthur, Treatment and Prevention of Decay of the Teeth, says. But it must be borne in mind, as has already been explained, that, after the surfaces of the dentine are exposed, in carrying out the treatment advised, some time is required for the consolidation of the fibrillæ cut through, and until this takes place decay is liable to recur, although not likely if the separated teeth do not again come in contact. At what time this change is completed can not be stated definitely. It may be in a few months, or not before the lapse of several years. This being true, then I say it is "nip and tuc," which does their work first calcification or Leptothrix Buccalis. So much has been said, of late, of the ravages of oral parasite, that I deem it unnecessary at this time to say more, that they are genuine dentos lovers and tube suckers I believe. If we could have the use of the ears of the microscope for a few moments, we would perhaps hear them (Leptothrix Buccalis) smacking their mouths over freshly cut dentine and clawing their way into the dentinal tubuli.

I read in the *Dental Cosmos*, of June, 1873, the following, "Tin in Teeth, by C. W. Foster." He has very wisely said. The probable cause of the preservative nature of tin in teeth is due most clearly to the aggregate qualities of the metal before spoken of, such as softness and adaptability, by which condition under pressure in the tooth cavity the metal will be found stopping the ends of the larger dentinal tubuli and interglobular spaces. He uses the oxide of tin, also known by the name of polishing powder, this powder, he applies to the inner walls of the tooth, for the purpose of effectually stopping the porous walls or tubes of the cavity.

Once more from the same writer, *Dental Cosmos*, March, 1873. "Gold in Teeth," he says we have for a long time considered that something which could be applied to the surface of the cavity previous to the introduction of the gold and used in the convenient and efficient form of a fluid, or semi-fluid, would be the most desirable, if at the same time, it met the demands of the case. The hypophosphite of lime which we had opportunities to use with perfect success, we have reasons to believe, seems to us to fulfill all the requisite conditions. Its application is easily made in fluid or semi-fluid state. It introduces into the ends of the tubuli a salt which is shortly transformed into phosphate of lime and so hastens the formation of the protecting and non-conducting envelope over the heretofore naked surface of the cavity, practically glazing and enameling the entire wall. What becomes of that portion of the tubes opening into the oral cavity. The tubes terminating in the cavity of a tooth are sealed, very closely sealed, but the other ends of the tubes are left open. Dr. E. Park, in an essay called Contour Fillings, read before the Missouri Dental Association, June, 1871, makes use of this argument, viz. "A better method to adopt in filling such teeth is one now advocated. After having filled the cavities, continue the operation until the contour of the teeth is restored, and the exposed dentine and enamel rods are perfectly protected.

Again, listen to one whose opinions, in a scientific sense, are respected by all true lovers of Dental Science. Tomes has truly and truthfully said, that the best fillings he ever saw, were those entirely surrounded by enamel. No one perhaps has given this subject more reflection and investigation in a scientific sense than he. Why is it, after all his research, does he come to the conclusion that the best fillings he ever saw, are entirely surrounded with enamel? The question is easily answered. In view of the above facts, I ask the question, where do we get the right or license or precedent to leave the dentinal tubes open, here it is, here is where we get it.

The Father of American dentistry, Dr. C. A. Harris, has said, "why is it that the negroes of Abyssinia have such sound teeth as they are represented to have, since it has long been a custom with them to file all their front teeth to points so as to make them resemble the teeth of a saw, or those of carniverous animals. Of course, large portions of the enamel and much of the bony structure, must be removed in the operation, yet we are credibly informed that their teeth seldom decay, the same may be said of other nations, the Brahmins, of India, who, from remote ages, have been in the habit of using the file principally, I believe, for separating their teeth, yet, they too, are noted for having fine teeth. He says further, the reason why their teeth are not so subject to disease as are those of the inhabitants of civilized countries, is attributable to the difference in their habits of life, mode of living and to the absence of the causes productive of the various diseases peculiar to civilization and refinement." The above argument may hold good with the natives of the torrid zone, but in this the temperate it will not, as past experience has proven.

Dr. Jno. Allen has remarked, that as a nation, we have worse teeth than any on the earth. Now why is this, simply because we change the proportions of these various constituents that our Creator has provided for us, by separating away what has been put there for the building up of the hard tissues. To prove this, let us look to other nations, for instance the Abyssinians, Mocoes, Mundingoes, of Africa, Brahmins of India, the Esquimax of the icy regions, and the Aborigines of the West. These tribes do not change the proportions of the various constituents (at all events they come nearer living up to them than we do) that enter into their bodies, do not have decayed teeth There is a constant change going on, and particles of matter are deposited atom by atom and the system kept fully charged with the mineral elements of which these structures are built up. When you look at nations, such as named above, that do not change the proportions you see no decayed teeth, and the history of these nations proves that their teeth are sound and beautiful to old age. Let us look at the other side of this subject. What is the condition of the race of people in the temperate zone. We do change these proportions, we do ignore the mineral elements provided for us, and we do have decayed teeth. I find that there are over twenty millions of teeth swept from our population every year. The prime cause of this is, we do not take the material into our system that is carried back atom by atom, and keep the hard tissues built up until the old particles pass away. The old particles pass away after they have served their purpose, and new ones then take their places.

From the foregoing conclusions, it is very plain to my mind, that these people differ from us in their entire organization, and too much so for us to be justified in drawing from them conclusions applicable to civilized people; yet we may deduce from these facts the conclusions that teeth well developed, will not suffer much from the free use of the file and exposure of the dentinal tubuli, while those of a less dense structure crumble away to dust.

It has been said by some fruitful mind, that teeth of Egyptian mummies have been found with gold in them; this I have not seen.

Again, it has been said, that the Abyssinians file their teeth to points, and the Mocoes and Mundingoes file groves in their teeth, and that they seldom decay; these are wonders which I never have seen. There is no question in my mind, that if these nations who file their teeth in such a manner as to leave the dentinal tubuli open, were subjected to the same influences that we are, that as a race of people, they would soon become edentulous.

Listen to Swift:

"Brutes find out where their talents lie;
A bear will not attempt to fly:
A foundered horse will oft debate,
Before he tries a five-barred gate:
A dog by instinct turns aside,
Who sees the ditch too deep and wide.
But man we find the only creature,
Who led by folly combats Nature,
Who when she loudly cries, forbear,
With obstinacy fixes there:
And where his genious least inclines
Absurdly bends his whole design."

Let us ask ourselves the questions. To what quarter are we drifting? Can we in these degenerate times leave the dentinal tubuli open? Are we not of the artificial—artificial? These are pertinent questions, and should be answered in truth and soberness. That we are in a great measure artificial, made so by our mode of living, is the well considered opinion of the wisest and ablest minds in the medical profession. This being true, I have felt it to be my duty to raise my feeble voice in no uncertain sound, to expose what I conceive to be a wrong.

The dentist who pooh-poohs when asked if exposure of the dentinal tubuli is no injury, is not in my opinion fully up with the times. To those who differ with me, I say, give me a precedent for such practice, of course, you would say that the Abyssinians and other nations file their teeth with impunity and that they seldom ever decay. Furthermore our grand fathers and grand mothers, many of whom are now living, had their teeth filed all to pieces when they were children, many of their teeth are good to-day. This argument may hold good, but still it does not prove directly, or remotely, that it is correct practice to leave the dentinal tubuli open.

Suppose our grand fathers and grand mothers, those of them that have the dental tubes open, should become match dippers and dyers, they would be fit subjects for that terrible disease called Phosphor-Necrosis, which never occurs except in persons affected with defective teeth. The disease evidently passes through the dental tubes into the pulp, and thus to the periosteum.

Is there no principal of vitality strong enough to defy at once assaults from without and disintegration from within. No elixir vitæ discovered by the accumulated sagacity, wisdom and experience of our faithful teachers in our noble and useful profession, to stay this immense loss of teeth when the enamel is removed. There may be a natural preventive, if so, I am not in possession of it, on the other hand I can say with some degree of certainty, that artificially we have an elixir vitae.

As I reach the conclusion of my subject, I can not but feel the inadequacy with which I have treated it, and in conclusion, permit me again to ask: Is it good practice to leave the dentinal tubuli open?

Artificial treatment, in all cases and under all circumstances, where the tooth or teeth as the case may be, is deprived of its enamel, enamel, so to speak, the entire surface of dentine and enamel rods with gold, thereby, hindering the passage of air, acids, fungus and animals in short all the destroying agents that the oral cavity is heir to.

